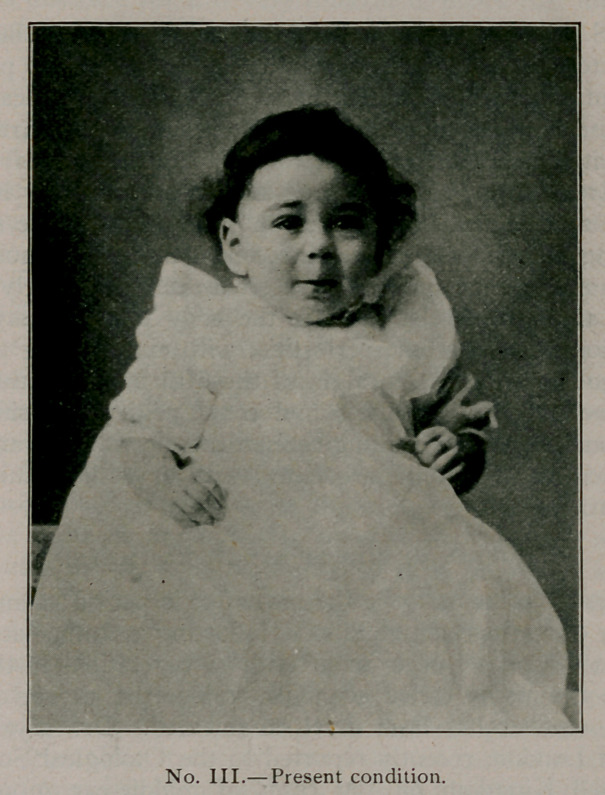# Two Cases of Special Interest1Presented to the Clinical Society of the New York Post-Graduate Medical School and Hospital, December 16, 1904.

**Published:** 1905-04

**Authors:** William Seaman Bainbridge

**Affiliations:** Surgeon New York Skin and Cancer Hospital; Attending Surgeon New York Children’s Hospitals and Schools; Adjunct Professor of Operative Surgery and Operative Gynecology, New York Post-Graduate Medical School and Hospital; Consultant New York Home for Destitute Crippled Children, New York


### Two Cases of Special Interest.[1]

### I.—Multiple Herniae. II.—Large Angioma Hypertrophicum.

By WILLIAM SEAMAN BAINBRIDGE, A. M., M. D.,

Surgeon New York Skin and Cancer Hospital; Attending Surgeon New York Children's Hospitals and Schools; Adjunct Professor of Operative Surgery and Operative Gynecology, New York Post-Graduate Medical School and Hospital; Consultant New York Home for Destitute Crippled Children, New York.

THE two cases herewith reported present a number of interesting and unusual conditions.

### I. A CASE OF MULTIPLE HERNIAE.

*History.*—Mary D., 45 years of age; married; three children, aged 23, 21 and 14.

*Family History.*—Negative. No herniæ.

*Previous Personal History.*—No serious illness. Menopause established one year ago. At thirty a small femoral swelling, about the size of the end of the index finger, came while patient was leaning over, and was marked by a feeling as if "something snapped." This small hernia gradually increased in size. When pregnant, the patient had a fulness at the navel which disappeared after each of the first two pregnancies, but persisted after the third, growing gradually larger. For some years there has been a very profuse, fetid, yellow leucorrheal discharge; bowels were normal. For many years the patient has had what she termed indigestion. Errors in diet at times caused the production of a great deal of gas, a feeling of weight in the stomach after eating, and discomfort usually until the bowels moved. During the past seven or eight years there have been repeated attacks of pain in the epigastrium, extending through to the back. These attacks came on rather suddenly and lasted often for several hours. At times the pain was so severe that she was obliged to pace the floor. The pain disappeared, usually, as suddenly as it came, and only some epigastric soreness and the general exhaustion from the suffering remained.

*Physical Examination.*—The following conditions were revealed upon examination: Umbilical hernia about the size of a large egg; chronic appendicitis; a benign ulcer of the cervix; retroversion and retroflexion of a large and flabby uterus; left femoral hernia the size of an orange.

*Operation.*—May 26, 1904, the patient was divulsed and curetted. Many fungosities were removed. The femoral hernia proved to be an epiplocele, and was treated by the Bassini method,

1. Presented to the Clinical Society of the New York Post-Graduate Medical School and Hospital, December 16, 1904.

kangaroo tendon being employed. Next, a free vertical incision was made in the middle line encircling the navel. The umbilical protrusion was found to be an entero-epiplocele, and directly

Drawing showing conditions found in central line of abdomen at time of operation:

1. Hernia epigastrica.

2. Umbilical entero-epiplocele.

3. Para-umbilical epiplocele.

4. Para-umbilical entero-epiplocele.

5. Lipoma with small epiplocele under neath.

above this, in the linea alba, was a small epiplocele about the size of the end of the thumb. Just below the umbilical hernia was an epiplocele, and below this again an entero-epiplocele, both being about the size of the one above the navel. Each hernia was a distinct protrusion through the aponeurosis into the thick fatty layer covering it. The overlapping of the aponeurotic layers (called by some, the Fowler-Mayo method,) was employed. Kangaroo tendon and chromicised catgut were used. Just below the hair line, in the centre, attached to the external surface of the peritoneum, was a small lipoma the size of a bird's egg which covered a slit in the linea alba through which protruded another small epiplocele. Before the incision in the fat was made only a very slight fulness could be detected in the central line of the abdomen, except at the umbilicus.

Before the closure of the abdominal wall the appendix was removed, and many adhesions in the neighborhood were tied off or cut away with the Paquelin cautery. Ventral fixation was now done, and the wound closed layer by layer. The condition of the patient at the end of the operation was very satisfactory. On June 8 the stitches were removed, and on June 29 patient was discharged from the hospital as cured. Since then there has been freedom from all symptoms. I would particularly note that there has been no return of the acute attacks of epigastric pain.

It is not uncommon for the recti muscles to be separated. Occasionally slits in the linea alba may be present. While these two conditions are well known to occur I fear, however, that not enough attention has been given to this form of herniæ. They are true protrusions of the contents of the abdomen, are difficult to diagnosticate, if small, and the patient is obese, and yet, as in the case reported for your consideration this evening, may cause acute attacks of pain which simulate a number of other conditions, such as biliary colic or gastric ulcer.

The patient has very kindly come over from Brooklyn tonight to be a visible demonstration of the complete cure.

Thus under one anesthetic the following operations were performed in this case: (1) divulsion and curettage; radical cure for: (2) femoral epiplocele; (3) hernia epigastrica; (4) umbilical entero-epiplocele; (5) para-umbilical epiplocele; (6) para-umbilical entero-epiplocele; (7) lipoma and epiplocele; (8) ventral fixation; (9) appendectomy.

### II. ANGIOMA OF FACE.

This case was referred to me, at the New York Skin and Cancer Hospital, by Dr. L. Duncan Bulkley. The photographs of the child show clearly the steps of the operation, as well as the final result obtained.

*History.*—Boy, 5 months old, only fairly well nourished.

*Family History.*—No history of angiomata or tumors of any kind. One other child, who is normal.

*Personal History.*—Breast-fed baby, perfect at birth. When two weeks old Dr. William McChristie, the attending physician, noticed a small blue spot under the skin on the upper lip just below the septum nasi. This grew rapidly and in a month was as large as a marble, forming quite a projection in the upper lip. At six

No. I.—Before the first radical operation.

weeks of age Dr. McChristie started the hot water injection treatment, introducing into the growth between an ounce and an ounce and a half of boiling water once a week. There was considerable sloughing after the second injection. Five successive treatments were given. For four weeks thereafter the growth remained apparently quiescent, but then began to grow rapidly.

*Operation.*—On May 6, 1904, operation under chloroform anesthesia. The tumor at that time, as shown in Picture No. 1, practically embraced all of the upper lip, extending well up around the alae nasi, and somewhat into the nasal cavity on the mucous membrane. There was an ulcerated surface in the centre which had lately healed. Extirpation of the growth was made as complete as possible. There was no limiting capsule apparent. Each ramification was followed and freely excised, extending the incision on to the septum in both nostrils, and around the alae nasi almost to the junction of the superior maxilla with the nasal bone on either side. A great deal of vascular and cicatricial tissue was removed. Hemorrhage was controlled by ligation of the coronary arteries *in situ.* Flaps from the adjacent tissue of the cheek were freed, and by means of catgut and silk sutures the parts were brought into apposition. There was considerable tension above,

No. II.—Taken before the second operation.

and for fear of possible sloughing it was deemed advisable to leave some redundancy of the mucous membrane of the lip at the vermilion border. The stitches held well despite the fact that after a few hours of crying breast feeding had to be commenced. The silk was removed on May 16.

Dr. J. C. Johnson, of Cornell University, examined the growth and reported it to be a "hypertrophic angioma."

Picture No. 2, taken in October, shows what was left of the redundant tissue. This had decreased in size somewhat since the operation, but the fulness of the mucous membrane was more everted. No apparent recurrence of the growth.

October 17 chloroform was again administered and the upper lip shaped by the removal of this small projection. Catgut and silk were employed and the latter removed on the tenth day.

Picture No. 3, taken a few days ago, gives the present condi-

No. III.—Present condition.

tion. A fine white line is all that remains to show what has been done. The lateral incisions are covered by the alae of the nose.

34 Gramercy Park.

---

Professor Postemski, of Rome, recently presented to the president of the Italian Red Cross Society the medical officers who had taken part in the campaign against malaria in the Campagna. The results have been highly satisfactory. The number of persons subjected to the prophylactic treatment with quinine was 11,962. Only 380 were attacked by the fever, of whom 67 had it as a primary and 313 as a relapsing disease. Some of the latter had suffered several times.—*Medical Age.*

## Figures and Tables

**Figure f1:**
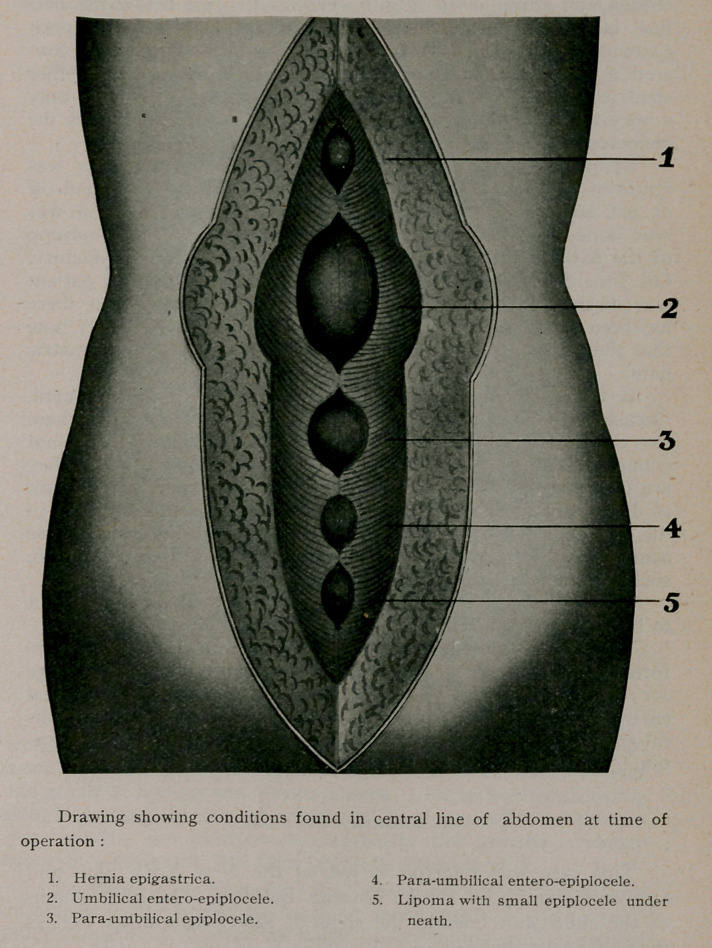


**No. I. f2:**
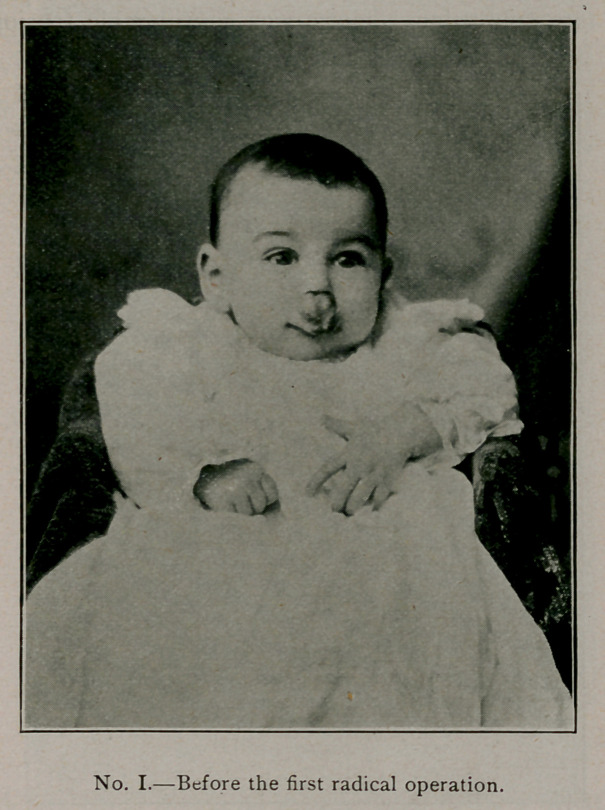


**No. II. f3:**
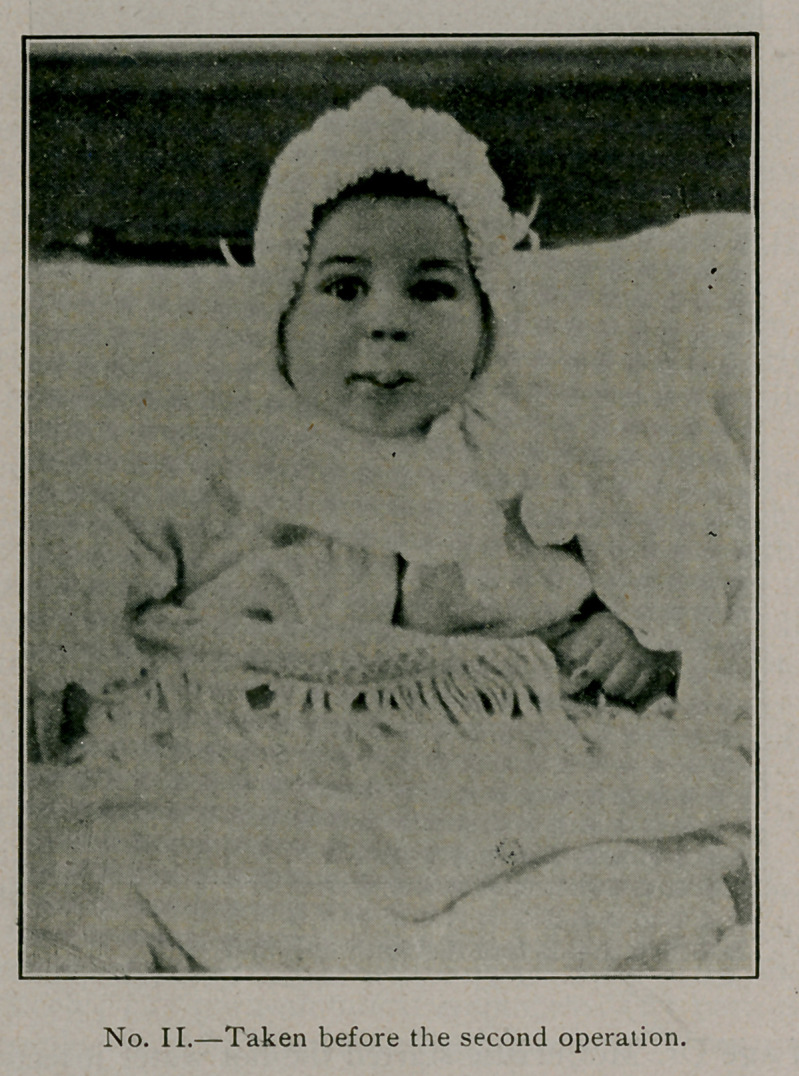


**No. III. f4:**